# Perspectives Regarding Mind Maps and Their Impact as a Self-Directed Learning Tool Among First-Year Undergraduate Bachelor of Medicine, Bachelor of Surgery (MBBS) Students

**DOI:** 10.7759/cureus.111811

**Published:** 2026-06-30

**Authors:** Neena Sharma, Prateek Bobhate

**Affiliations:** 1 Department of Physiology, All India Institute of Medical Sciences (AIIMS) Jammu, Jammu, IND; 2 Department of Community Medicine, All India Institute of Medical Sciences (AIIMS) Jammu, Jammu, IND

**Keywords:** medical education, mind mapping, physiology, self-directed learning, undergraduate medical students

## Abstract

Background: Mind mapping is a visual, non-linear learning technique in which information is organized around a central concept with related topics and subtopics arranged as branches. It promotes active learning by helping students organize, understand, and recall information. In medical education, where students are required to learn large volumes of conceptual content, mind mapping may serve as a useful self-directed learning tool.

Aim: This study aimed to evaluate the effectiveness of mind mapping as a self-directed learning method among first-year Bachelor of Medicine, Bachelor of Surgery (MBBS) students and to assess students' perceptions regarding its use in the context of physiology.

Materials and methods: This quasi-experimental, non-randomized controlled crossover study was conducted among 100 first-year MBBS students at All India Institute of Medical Sciences (AIIMS) Jammu in Jammu, India. Students were divided into two routine practical batches of 50 each. In Phase 1, Batch A served as the mind mapping group and Batch B as the traditional learning group for the topic "Acid-Base Disorders". All students were first trained in mind map preparation using a topic unrelated to the study intervention. Students in the mind mapping group studied the assigned topic by preparing mind maps, while students in the traditional learning group used their usual study methods. Immediate and delayed post-tests were conducted, with the delayed test performed one month after the intervention. In Phase 2, crossover was performed using the topic "Physiology of EEG and Clinical Relevance", and the same process was repeated. At the end of the study, students' perceptions were assessed using a structured five-point Likert scale questionnaire. Data were analyzed using IBM SPSS Statistics for Windows, Version 15.0 (SPSS Inc., Chicago, Illinois, United States), and a p-value of ≤0.05 was considered statistically significant.

Results: Immediate assessment scores did not differ significantly between the mind mapping and traditional learning groups in either phase or in the combined analysis. In the delayed assessment for Phase 2, the mind mapping group scored significantly higher than the traditional learning group. The combined delayed assessment analysis also showed significantly higher scores in the mind mapping group compared with the traditional learning group, with 100 observations in each group (6.97±1.97 vs. 6.30±2.28; p=0.029; Cohen's d=0.313). Student perception was favorable, with most students reporting that mind mapping improved understanding, organization of concepts, engagement, confidence, active participation, and independent learning. These findings suggest that mind mapping was well accepted by students as a self-directed learning strategy and may contribute positively to independent learning, engagement, and the overall learning experience in undergraduate medical education.

Conclusion: Mind mapping did not significantly improve immediate assessment performance compared with traditional learning; however, it was associated with better delayed assessment scores, suggesting a potential benefit in long-term retention. Students perceived mind mapping as an engaging and useful self-directed learning tool. Mind mapping may be incorporated as an adjunct to traditional teaching-learning methods in undergraduate medical education.

## Introduction

A brainstorming strategy that presents information in a hierarchical structure is known as mind mapping. It's a strong graphic method that can be used to boost learning and encourage better thought [1]. The idea of mind maps is to help people understand, remember, and enjoy what they learn better, since they use pictures to make their understanding better [2]. Physiology is one of the core subjects that is taught in the first year of undergraduate medical courses. The physiological teaching-learning process consists largely of lectures, tutorials, and practical sessions, which are carried out traditionally [3]. These techniques have been the main way of teaching since the beginning of the formal education of medicine. Traditional teaching is mainly one-way communication; students listen to teachers and take notes. While traditional teaching methods give students a clear structure and enable efficient information transfer, they can also restrict students' interaction with the material, which may result in disengagement and less retention of information. In the era of teaching by telling, or teacher-centric learning, it is now being challenged by more student-centric learning approaches, which are more in line with the constructivist learning approach [3]. Medical students are introduced to lots of information in their undergraduate studies. This information can be difficult for students to organize and remember. Consequently, they can be passive receivers of a lot of information that is sent to them by the teachers, and to a lesser degree, they would participate in the learning process [4].

Active learning techniques encourage students to do things that stimulate deep learning, reflection, and assimilation of information. Active learning is generally defined as any instructional method that actively involves students in the learning process [5]. It is intended to engage students in a more active participation than conventional teaching and to get them involved in activities other than listening [6]. The shift from teacher-centered to student-centered learning is increasing in medical education. Student-centered approaches encourage brainstorming, idea generation, critical and analytical thinking, problem-solving, decision-making, and active engagement in the learning process. Such approaches help learners construct knowledge independently and develop lifelong learning skills that are essential for medical practice [7]. Additionally, visual learning tools can help to lower stress and information overload and improve communication and clarity, since the visual representation is generally more interesting. As an active learning tool, the use of mind maps can thus be an innovative approach to improve student learning [8]. The concept behind mind mapping is the idea of radiant thinking, and the inspirations of mind mapping can be traced back to Leonardo da Vinci's notebooks. Mind mapping was popularized worldwide in the 1960s by British psychology author and education consultant Tony Buzan. Mind maps are used in various activities and by professionals across different fields. In the field of education, they can be used as instruments to help and improve the learning process. A mind map has been identified as a visual tool that transforms information and knowledge into a hierarchical structure of structured key terms, images, and associations [9].

Mind maps are created in a similar way to a tree, having one point at the center with main topics and subtopics branching out like branches and twigs. A diagram is a series of words, short phrases, concepts, or things that relate to a central concept and are jumbled around it. The information is connected through these ideas, so that it is easier to remember and understand [10]. In this way, the information which is initially encoded in a text passage is hierarchically organized, with the more general information in the center and the more detailed information at the periphery. This structured way of visualizing information can help to remember, reinforce, and enhance communication. Therefore, the mind mapping method can be said to be a learning tool that helps to improve learning, retention, and understanding [11]. The key aspects of mind mapping include starting with a main topic, creating a central image or idea, creating subtopics, linking related ideas, drawing twigs to a less important topic, and adding information and detail to each topic. These features can be used to present information in an organized and structured way that may help understand and recall information [12].

Mind maps have been a part of education for many years. The literature, however, shows conflicting results in terms of immediate assessment test scores in medical education after a mind map teaching method. Another level of scientific validation is that mind mapping as a tool for education is not consistent [13]. Furthermore, very few studies have examined the scores of the delayed post-test for mind mapping versus conventional learning approaches [14]. What's more, students' acceptance of mind mapping in medical teaching has been reported as being low in previous studies [15]. Therefore, the present study was performed to examine the effectiveness and long-term educational benefits of mind mapping in the teaching-learning process and students' attitudes towards using mind mapping.

## Materials and methods

Study design

This study was conducted as a quasi-experimental, non-randomized controlled study with a crossover design to assess the impact of mind mapping as a self-directed learning tool among first-year undergraduate Bachelor of Medicine, Bachelor of Surgery (MBBS) students.

Study setting

The study was conducted in the Department of Physiology of All India Institute of Medical Sciences (AIIMS) Jammu in Jammu, India, among first-year undergraduate MBBS students of the 2024 batch.

Study duration

The study was conducted over a period of five months. It included an initial mind mapping training session, Phase 1 intervention and immediate assessment, a delayed assessment conducted one month after Phase 1, Phase 2 crossover intervention and immediate assessment, a delayed assessment conducted one month after Phase 2, and final administration of the student feedback questionnaire. Each intervention session was conducted during routine scheduled teaching hours, and both groups were allotted equivalent study time.

Study population and sample size

The study population comprised 100 first-year undergraduate MBBS students from the 2024 batch. All students in the batch were considered for participation in the study. Since the entire available batch was included, universal sampling was used.

Ethical considerations

Approval was obtained from the Institute Ethics Committee of AIIMS Jammu (approval number: AIIMS/Jmu/IEC/2025/36). Before the start of the study, students were given background information about the study's purpose and procedure. The learning activity was an integral part of the regular educational and evaluation process. For research analysis, only anonymized data were used. Students' assessment scores and feedback responses were used for research purposes with their consent.

Eligibility criteria

Inclusion Criteria

All first-year undergraduate MBBS students of the 2024 batch who participated in the teaching-learning sessions and assessments were included in the study.

Exclusion Criteria

There is no exclusion criterion, as participation in the study and mind map creation is considered part of the internal assessment activity.

Group allocation

This batch of 100 students was split into two routine practical batches, 50 students in each. Batch A consists of students with roll numbers ranging from 1 to 50, while Batch B consists of students with roll numbers ranging from 51 to 100. These were regular practical batches, which turned over between physiology and biochemistry practicals. Groups were not randomized, but were based on existing academic batch allocation. The first phase was to have Batch A as the mind mapping group and Batch B as the traditional learning group.

Training on mind mapping

Before the intervention, all students attended a training session on the mind mapping technique as part of their normal teaching time. This session was meant to give the students an introduction to the concept, structure, and use of mind maps as a learning tool. For training purposes, a text on "Autonomic Nervous System" was chosen from "Guyton and Hall Textbook of Medical Physiology", 5th South Asian Edition. This topic was only introduced to show how mind maps were created and used and did not relate to the topics for study that were assessed. The training session began with a slide presentation that explained the concept of mind maps, the elements of mind maps, and how to create a mind map. Students were provided with the opportunity to ask questions about the technique. As a result of the training session, each student was required to create at least one mind map to get basic knowledge about mind mapping.

First phase of intervention

Both groups were exposed to the study text on "Acid-Base Disorders" after the training in their routine teaching hours. The topic was chosen to avoid the researcher's preconception having any effect on the results of the study. The intervention was conducted during regular scheduled teaching sessions, and both groups were allotted equivalent study time. In the mind mapping group, students were asked to allocate their study time between reading the text they were assigned, making a mind map, and then revising the topic based on the mind map. The students in the traditional learning group were taught to learn in their own ways by reading the assigned text. During this stage, students in a traditional learning group were not guided to create mind maps. A scoring method was proposed to evaluate the students' mind maps, taking into account the structure and content of the mind map. The mind maps were evaluated for academic encouragement using a simple scoring approach based on inclusion of the central topic, appropriate branching of major and minor concepts, logical organization, clarity of presentation, and completeness of relevant content. These scores were used only for student encouragement and felicitation and were not included in the statistical analysis; therefore, they had no influence on the study findings.

Assessment of learning outcomes

Following the study session, all students were assessed using an immediate assessment test. A delayed assessment was conducted one month after the intervention to assess retention of knowledge. Each test consisted of 10 multiple-choice questions (MCQ), with a maximum score of 10. The assessment questionnaires used for immediate and delayed post-tests in both intervention phases are provided in Appendix A, Appendix B, Appendix C, and Appendix D.

Crossover phase

A crossover was conducted to further verify the effectiveness of mind mapping and minimize any bias seen in the group. In the crossover stage, Batch A was the traditional learning group, and Batch B was the mind mapping group. The chosen topic for the crossover phase was "Physiology of EEG and Clinical Relevance". The same intervention procedure was followed. The mind mapping group used mind mapping to study the topic, and the traditional learning group used the traditional method of study. The immediate and delayed post-test scores were taken and contrasted for the two learning strategies.

Student feedback and perception assessment

A structured feedback questionnaire was used to assess the students' perceptions about mind mapping as a learning tool in physiology at the end of the study. The questions in the questionnaire were rated on a five-point Likert scale from strongly agree to strongly disagree. Also included in the questionnaire were relevant information regarding the demographic and academic information of the participants. At the end of the study, students' perceptions regarding mind mapping as a self-directed learning tool were measured using a structured 10-item questionnaire rated on a five-point Likert scale. The complete feedback questionnaire is provided in Appendix E. The questionnaire was rated with a five-point Likert scale from strongly disagree to strongly agree. The questionnaire measured the students' attitude towards understanding, involvement, conceptual organization, active participation, confidence, enjoyment, independent learning, time spent on the study, exam preparation, and students' plans to use mind maps. Results for each of the items in the full questionnaire and the distribution of responses to each item are reported in the Results section. 

Questionnaire development and validation

The feedback questionnaire was designed from the literature and adapted to the aims of this study. It consisted of 10 items related to students' perceptions of mind mapping's usefulness in understanding concepts, organizing information, enhancing memory and recall, stimulating active learning, preventing information overload, generating interest in physiology, facilitating revision, promoting self-directed learning, and willingness to continue using mind maps as a learning tool. The questionnaire was created out of the present literature and amended as per the aims of the present study. Before administration, the questionnaire underwent expert review by internal and external subject specialists as well as a medical education expert to assess relevance, clarity, comprehensibility, and alignment with the study objectives. Minor wording modifications were made based on expert suggestions. Although formal reliability testing, such as Cronbach's alpha estimation, was not undertaken, content validity was established through this expert evaluation process. This limitation has been acknowledged in the revised manuscript.

Statistical analysis

The analyses of the data were performed using IBM SPSS Statistics for Windows, Version 15.0 (SPSS Inc., Chicago, Illinois, United States). Data for continuous variables, such as immediate and delayed assessment scores, were presented as mean and standard deviation (SD). To compare the post-test scores of students' "traditional method" with those of "mind mapping", an independent samples t-test was used. A five-point Likert scale questionnaire was used to determine students' perception of mind mapping and was presented in the form of frequencies and percentages. P-values of ≤0.05 were considered statistically significant.

## Results

In this quasi-experimental, non-randomized controlled crossover study, 100 students of the first MBBS batch of 2024 participated. Based on the present institutional practical class allocation, students were split into two batches of 50 students each. The students in Batch A had roll numbers from 1 to 50, and in Batch B, they had roll numbers from 51 to 100. For the topic "Acid-Base Disorders", Batch A was the mind mapping group and Batch B was the traditional learning group in Phase 1. For the topic "Physiology of EEG and Clinical Relevance" in Phase 2, a crossover was done with Batch B as the mind mapping group and Batch A as the traditional learning group. Data from all 100 students were available for Phase 1. In Phase 2, 50 students in the mind mapping group and 50 students in the traditional learning group had both immediate and delayed assessment data. Thus, the combined analysis involved 100 observations for the mind mapping group and 100 observations for the traditional learning group.

Phase 1: "Acid-Base Disorders"

*Immediate Assessment* *Scores*

In Phase 1, the mean immediate assessment score was 6.24±1.91 in the mind mapping group and 6.32±1.94 in the traditional learning group. The difference between the two groups was not statistically significant. Table 1 shows no significant difference in immediate scores.

**Table 1:** Comparison of immediate assessment scores for "Acid-Base Disorders" between the mind mapping and traditional learning groups

Assessment	Group	n	Mean±SD	t-value	P-value	Result
Immediate assessment	Mind mapping	50	6.24±1.91	-0.208	0.836	Not significant
Immediate assessment	Traditional learning	50	6.32±1.94			

Delayed Assessment Scores

In the delayed post-test conducted one month after the intervention, the mean score was 6.26±1.90 in the mind mapping group and 5.84±2.33 in the traditional learning group. The difference between the two groups was not statistically significant. Table 2 shows no significant difference in delayed scores.

**Table 2:** Comparison of delayed assessment scores for "Acid-Base Disorders" between the mind mapping and traditional learning groups

Assessment	Group	n	Mean±SD	t-value	P-value	Result
Delayed assessment	Mind mapping	50	6.26±1.90	0.986	0.326	Not significant
Delayed assessment	Traditional learning	50	5.84±2.33			

Phase 2: "Physiology of EEG and Clinical Relevance"

Immediate Assessment Scores

In Phase 2, the mean immediate assessment score was 7.14±2.29 in the mind mapping group and 7.86±1.58 in the traditional learning group. The difference between the two groups was not statistically significant. Table 3 shows no significant difference in immediate scores.

**Table 3:** Comparison of immediate assessment scores for "Physiology of EEG and Clinical Relevance" between the mind mapping and traditional learning groups

Assessment	Group	n	Mean±SD	t-value	P-value	Result
Immediate assessment	Mind mapping	50	7.14±2.29	-1.807	0.074	Not significant
Immediate assessment	Traditional learning	50	7.86±1.58			

Delayed Assessment Scores

In the delayed assessment conducted one month after the intervention, the mean score was 7.68±1.79 in the mind mapping group and 6.78±2.15 in the traditional learning group. The difference between the two groups was statistically significant. Table 4 shows significantly higher delayed scores in the mind mapping group.

**Table 4:** Comparison of delayed assessment scores for "Physiology of EEG and Clinical Relevance" between the mind mapping and traditional learning groups *p<0.05 was considered statistically significant.

Assessment	Group	n	Mean±SD	t-value	P-value	Effect size	Result
Delayed assessment	Mind mapping	50	7.68±1.79	2.275	0.025*	Cohen's d=0.457	Statistically significant
Delayed assessment	Traditional learning	50	6.78±2.15				

Combined analysis of both topics

Immediate Assessment Scores

In the combined analysis of immediate assessment scores across both topics, the mean score was 6.69±2.15 in the mind mapping group and 7.08±1.93 in the traditional learning group. The difference between the groups was not statistically significant. Table 5 shows no significant difference in combined immediate scores.

**Table 5:** Combined analysis of immediate assessment scores across both topics

Assessment	Group	n	Mean±SD	t-value	P-value	Result
Immediate assessment	Mind mapping	100	6.69±2.15	-1.350	0.178	Not significant
Immediate assessment	Traditional learning	100	7.08±1.93			

Delayed Assessment Scores

In the combined analysis of delayed assessment scores across both topics, the mean score was 6.97±1.97 in the mind mapping group and 6.30±2.28 in the traditional learning group. The difference between the groups was statistically significant. Table 6 shows significantly higher combined delayed scores in the mind mapping group.

**Table 6:** Combined analysis of delayed assessment scores across both topics p < 0.05 was considered statistically significant.

Assessment	Group	n	Mean±SD	t-value	P-value	Effect size	Result
Delayed assessment	Mind mapping	100	6.97±1.97	2.206	0.029*	Cohen's d=0.313	Statistically significant
Delayed assessment	Traditional learning	100	6.30±2.28				

**Figure 1:**
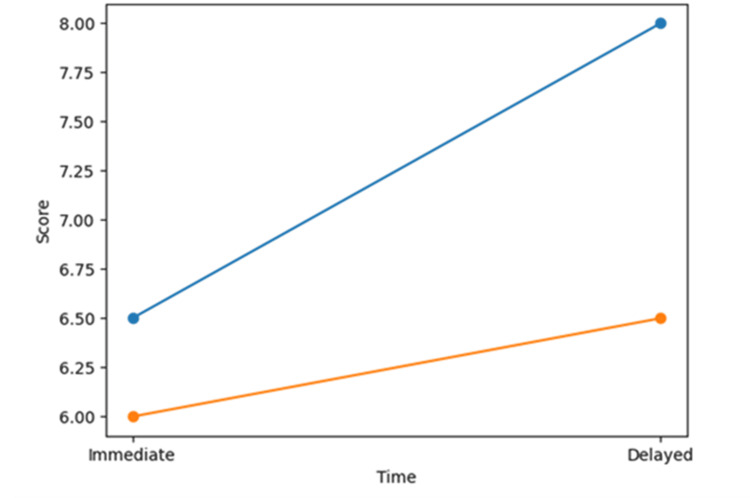
Trend of knowledge retention over time in the mind mapping and traditional learning groups

The trend of knowledge retention over time is depicted in Figure 1.

Score distribution in the combined delayed assessment 

In the combined delayed assessment analysis, 69 of 100 observations (69%) in the mind mapping group scored 7 or above out of 10, compared with 52 of 100 observations (52%) in the traditional learning group. Similarly, 43 of 100 observations (43%) in the mind mapping group scored 8 or above out of 10, compared with 31 of 100 observations (31%) in the traditional learning group. Table 7 shows a higher score distribution in the mind mapping group.

**Table 7:** Score distribution in the combined delayed assessment​​​​​​​ Values are presented as n/N (%). Each group included 100 combined observations across the two study phases.

Score band	Mind mapping group	Traditional learning group
Scored 7 or above out of 10	69/100 (69%)	52/100 (52%)
Scored 8 or above out of 10	43/100 (43%)	31/100 (31%)

Student perception of mind mapping

Students' perceptions regarding mind mapping as a self-directed learning tool were assessed using a five-point Likert scale questionnaire. Responses were summarized as "agree/strongly agree", "neutral", and "disagree/strongly disagree". Overall, student responses toward mind mapping were favorable. The highest positive response was observed for the statement "Mind maps helped me organize physiology concepts better", with 90% of students agreeing or strongly agreeing. Positive responses were also observed for improved understanding of physiology concepts, engagement with lecture content, active participation, confidence, and independent learning. Table 8 shows favorable student perceptions toward mind mapping.

**Table 8:** Student perception of mind mapping as a self-directed learning tool Responses for all statements were obtained from 100 students.

No.	Statement	Agree+strongly agree (%)	Neutral (%)	Disagree+strongly disagree (%)
1	Using mind maps improved my understanding of physiology concepts	87	11	2
2	I felt more engaged in the lecture content when making mind maps	85	15	0
3	Mind maps helped me organize physiology concepts better	90	9	1
4	Preparing mind maps encouraged active participation in learning	82	18	0
5	Using mind maps made me feel more confident about my knowledge of the lecture material	80	18	2
6	Mind maps made the learning experience more enjoyable	79	20	1
7	Mind maps encouraged me to engage in independent learning outside of lectures	78	18	4
8	Using mind maps reduced the time I needed to study and understand physiology topics	81	18	1
9	Mind maps were an effective tool for preparing for tests and exams	77.8	22.2	0
10	I would likely use mind maps in subjects outside of physiology for similar benefits	83.8	15.2	1

**Figure 2:**
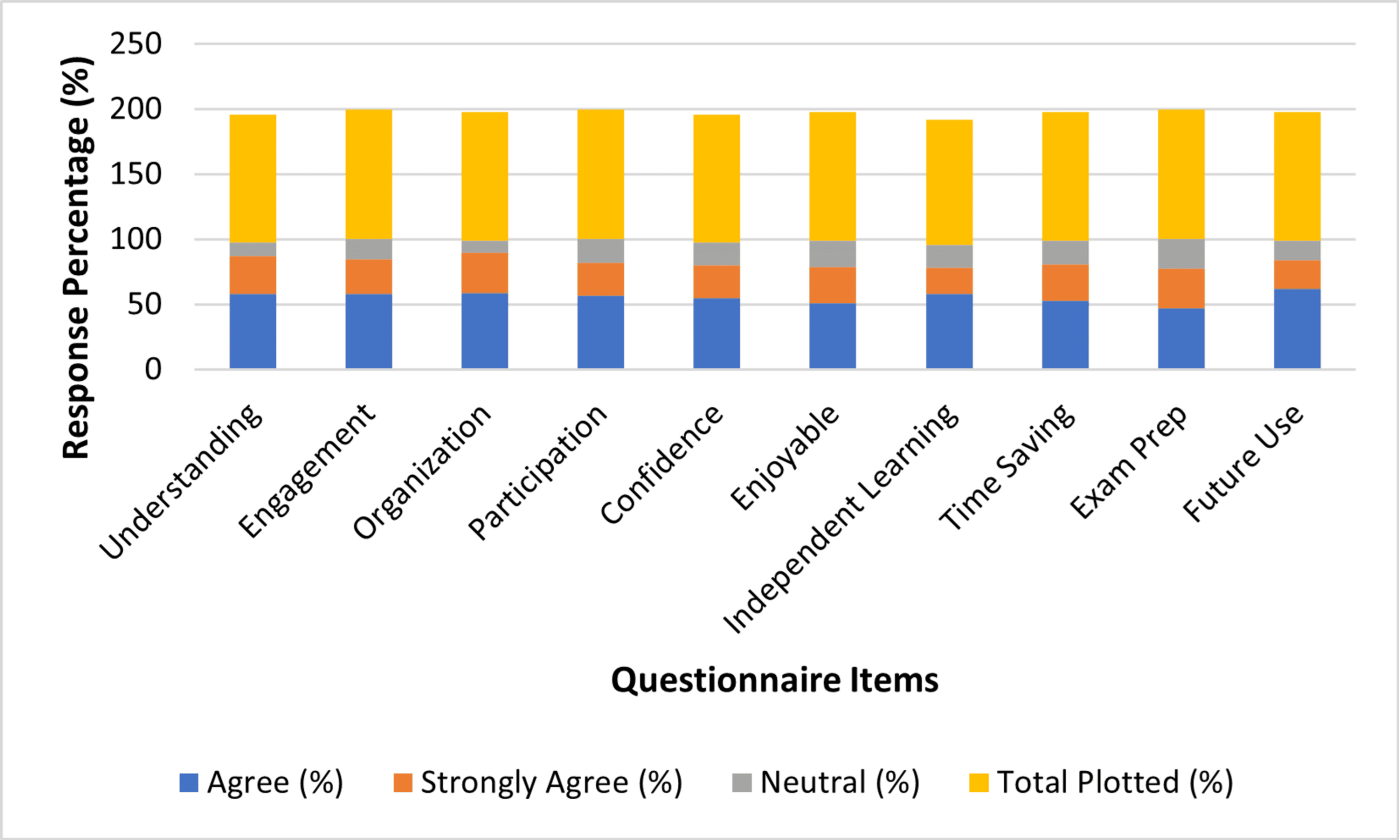
Distribution of student responses regarding mind mapping as a self-directed learning tool

Figure 2 shows the percentage distribution of responses across Likert scale categories for each questionnaire item.

Summary of findings

There was no significant difference between the mind mapping and traditional learning groups in the immediate assessment​​​​​​​ scores in either phase or combined analysis. In Phase 2, the combined analysis scores on the delayed assessment​​​​​​​ were significantly higher in the mind mapping group. The student perception responses indicated that most students had a positive self-directed learning experience by using mind mapping.

## Discussion

This quasi-experimental, non-randomized controlled crossover study was conducted among 100 first-year MBBS students at AIIMS Jammu to assess the effect of mind mapping as a self-directed learning strategy in physiology and to explore students' perception about mind mapping. A crossover design was used to minimize differences between the two batches related to the fixed group to ensure that both batches experienced both methods of learning (mind mapping and traditional) on two topics [16]. The crossover design, detailed description of the interventions, clearly defined assessment procedures, and inclusion of the assessment and feedback questionnaires in the Appendices support reproducibility and may facilitate future studies comparing mind mapping with traditional learning methods in medical education.

The mind mapping and traditional learning groups were found to be comparable in both phases and in the overall (combined) analysis in the immediate assessment. In "Acid-Base Disorders", the mean score of the mind mapping group was 6.24±1.91, and the traditional learning group was 6.32±1.94. The mean score of the mind mapping group was 7.14±2.29, while that of the traditional learning group was 7.86±1.58 for the "Physiology of EEG and Clinical Relevance". The immediate assessment test analysis also revealed no significant difference when the data from all the tests were combined. The results indicate that the advantage of mind mapping over conventional learning in terms of immediate measurable gains for short-term recall is not obvious. The differences between the scores of the mind mapping and traditional learning groups were not significant in the immediate assessment [17,18].

That not much has been gained in immediate scores could be associated with the type of short-term recall. Students may perform at the same level in an MCQ-based assessment, immediately after teaching, with both methods. The use of traditional reading, note-taking, and revising approaches might suffice for immediate recall. Mind mapping, however, is a process that involves active use, identification of key concepts, hierarchy, and connection of related ideas. The processes may not result in an immediate score increase, but may help in the deeper encoding of the information and later retrieval [19,20].

The absence of a significant difference in immediate assessment scores suggests that mind mapping may not provide an immediate advantage over traditional learning methods in short-term recall-based assessments. However, the significantly higher delayed assessment scores in the mind mapping group for the topic "Physiology of EEG and Clinical Relevance" in Phase 2 indicate better long-term retention among students who used mind mapping compared with those who used traditional learning methods. The scores of the mind mapping group were also significantly higher on the combined delayed assessment analysis, with 100 observations in each group. The delayed assessment showed a larger percentage of students from the mind mapping group who scored 7 and 8, respectively, in the composite tests, which was better than the traditional learning group. These results suggest that mind mapping may have a more significant impact on long-term memory than on short-term memory [20,21]. The mind mapping activity and visualization might account for the improved delayed performance. Students are asked to analyze the topic, identify the central concept, organize subtopics, and make connections between ideas when creating a mind map. It transforms linear information into a structured visual format, which can enhance understanding, associative memory, and recall [20,22]. Physiology is defined as the mechanisms, pathways, and interrelated concepts. Mind mapping could be useful in organizing and retaining this information [16].

The students' perception of mind mapping was positive. The majority of students agreed or strongly agreed that mind maps improved their understanding and organization of physiology concepts, promoted engagement and active participation, increased confidence, reduced study time, and supported self-directed learning. The greatest positive response was for improved organization of physiology concepts. This finding is important because effective organization of information is essential for understanding and retaining the large volume of knowledge encountered during the first year of medical education. The results obtained are similar to Kaup et al. [10], who found positive student feedback on retention and time management. The students' eagerness to apply the mind maps in other subjects also showed that they felt that mind maps were practical and useful for other subjects [17,23]. The use of mind mapping could enable students to move away from receiving information and towards constructing knowledge. This is in line with competency-based medical education, learning by doing, self-directed learning, and integration of concepts [23,24]. Student mind maps can also be useful in the eyes of the faculty to detect the conceptual clarity, misconceptions, gaps in knowledge organization, etc.

Strengths

The strengths of this study include its quasi-experimental crossover design, inclusion of all 100 first-year MBBS students from the study batch, assessment of both immediate and delayed learning outcomes, and evaluation of students' perceptions in addition to test scores. The intervention was also directly relevant to undergraduate medical education because it was conducted using physiology topics within the regular teaching-learning context. The crossover approach allowed both student batches to experience mind mapping and traditional learning, thereby reducing fixed batch-related differences. The findings suggest that mind mapping may support better delayed retention and may be a useful adjunct to conventional learning methods.

Limitations and future directions

This study has certain limitations. It was a non-randomized study, with group allocation based on existing practical class batches. The crossover design helped minimize group-related differences; however, random allocation was not possible. The study was conducted at a single institute among one batch of first-year MBBS students; therefore, the findings may not be generalizable to other institutes or student batches. Only two physiology topics were included, so the results may not be transferable to other physiology topics or other medical subjects. The assessment consisted of MCQ, which primarily tested recall of knowledge and may not have fully assessed higher-order cognitive skills such as analytical thinking, creative thinking, problem-solving, and clinical reasoning. Student perception was subjective and may have been influenced by response bias. The feedback questionnaire was reviewed for content validity by subject and medical education experts; however, formal reliability testing was not performed, which should be considered while interpreting the perception-related findings. Delayed retention was assessed only after one month and was not evaluated further. As the present study was not designed to directly evaluate higher-order cognitive domains such as critical thinking, clinical reasoning, or problem-solving skills, the conclusions have been restricted to outcomes that were objectively measured and supported by the data.

Future studies should include larger sample sizes, randomized designs, and multicentric involvement to strengthen the evidence and improve generalizability. Although the present findings suggest that mind mapping may promote deeper processing of information and improved retention, additional research using appropriate outcome measures is required to establish its effects on higher-order cognitive functions. The impact of mind mapping should be further studied with respect to long-term retention, conceptual integration, clinical application of knowledge, problem-solving skills, and academic achievement across different stages of medical education. Future studies should also include formal psychometric validation and reliability testing of feedback instruments used to assess student perceptions. Comparative studies of hand-drawn and digital mind maps may also be useful. Overall, mind mapping may be considered a valuable adjunct to conventional learning approaches in first-year medical education, as it was associated with higher delayed assessment scores and positive student perceptions, despite the absence of significant improvement in immediate assessment scores.

## Conclusions

This study indicates that mind mapping may be a useful supplementary technique to conventional learning among first-year MBBS students. There was no significant difference in immediate assessment performance between the mind mapping and traditional learning groups; however, delayed assessment scores after one month, particularly in Phase 2 and in the combined analysis, were significantly higher among students who used mind mapping, suggesting a potential benefit for long-term retention of physiology concepts rather than immediate recall. Students' perceptions were favorable, with most students reporting that mind mapping helped them understand concepts better, organize information, remain engaged, gain confidence, participate actively, learn independently, and prepare for examinations. Taken together, the crossover study design, objective assessment outcomes, and positive student feedback support the use of mind mapping as a student-centered, self-directed complementary learning technique in undergraduate medical education that may enhance, rather than replace, conventional teaching methods.

## Appendices

Appendix A

The questionnaires used for immediate and delayed assessments during both phases of the intervention, along with the structured feedback questionnaire assessing students' perceptions of mind mapping, are provided in Appendix A.

First Phase Intervention: Immediate Assessment (10 MCQ)

Q1. Primary buffer of extracellular fluid (ECF) is:

A. Hemoglobin B. Phosphate buffer C. Bicarbonate buffer D. Protein buffer

Q2. The most important intracellular buffer is:

A. Bicarbonate B. Phosphate C. Hemoglobin D. Lactate

Q3. Henderson-Hasselbalch equation relates pH to:

A. Osmolarity and CO₂ B. Ratio of base to acid C. BUN and creatinine D. Na⁺ and K⁺

Q4. pH 7.18, HCO₃⁻ 12, PCO₂ 22 mmHg. What is the disorder?

A. Metabolic acidosis with inadequate compensation B. Metabolic acidosis with respiratory compensation C. Mixed metabolic + respiratory acidosis D. Respiratory acidosis partially compensated

Q5. The strongest buffer in plasma at physiological pH is:

A. HCO₃⁻ B. Albumin C. Carbonic acid D. Phosphate

Q6. Which enzyme drives bicarbonate generation in kidneys?

A. Na⁺/H⁺ ATPase B. Carbonic anhydrase C. Alpha-ketoglutarate reductase D. Renin

Q7. High altitude causes respiratory alkalosis due to:

A. ↓ O₂ → ↓ H⁺ B. ↑ O₂ → ↑ H⁺ C. ↓ O₂ → ↑ ventilation D. ↑ CO₂ → ↓ ventilation

Q8. What is the best index of renal acid excretion?

A. Titratable acid B. Plasma HCO₃⁻ C. Urinary NH₄⁺ D. Urinary pH

Q9. Why is phosphate a poor ECF buffer?

A. Solubility issues B. Low concentration in ECF C. Cannot bind H⁺ D. Wrong pKa

Q10. A patient has PCO₂ 60 mmHg, HCO₃⁻ 35 mEq/L, pH 7.36. Diagnosis?

A. Acute respiratory acidosis B. Chronic respiratory acidosis with compensation C. Metabolic alkalosis D. Mixed disorder

Appendix B

The delayed assessment questionnaire, used one month after the first phase intervention, is provided in Appendix B.

First Phase Intervention: Delayed Assessment (10 MCQ)

1. A patient has PCO₂ 60 mmHg, HCO₃⁻ 35 mEq/L, pH 7.36. Diagnosis?

A. Acute respiratory acidosis B. Chronic respiratory acidosis with compensation C. Metabolic alkalosis D. Mixed disorder

2. The normal ratio of bicarbonate to carbonic acid (HCO₃⁻:H₂CO₃) in arterial blood is approximately:

A. 1:10 B. 10:1 C. 20:1 D. 5:1

3. The most important intracellular buffer is:

A. Bicarbonate B. Phosphate C. Hemoglobin D. Lactate

4. In metabolic acidosis, the kidney compensates by:

A. Decreasing H⁺ secretion B. Increasing HCO₃⁻ excretion C. Increasing NH₃ formation D. Decreasing NH₄⁺ secretion

5. Normal anion gap is:

A. 2-4 B. 4-8 C. 8-12 D. 18-24

6. A patient with pH 7.58, PCO₂ 30 mmHg has:

A. Metabolic acidosis B. Respiratory alkalosis C. Respiratory acidosis D. Metabolic alkalosis

7. Which is a cause of respiratory acidosis?

A. Hyperventilation B. Salicylate poisoning C. COPD D. High altitude

8. High altitude causes:

A. Respiratory acidosis B. Respiratory alkalosis C. Metabolic acidosis D. Metabolic alkalosis

9. Which buffer acts fastest?

A. Bicarbonate B. Hemoglobin C. Phosphate D. Respiratory mechanism

10. Maximum buffering capacity inside cells is due to:

A. Proteins B. Chloride C. Lactate D. Sulfate

Appendix C

The assessment questionnaire used during the crossover intervention is provided in Appendix C.

Crossover Intervention: Immediate Assessment (10 MCQ)

1. EEG primarily records which type of neuronal activity?

A. Action potentials B. Summated postsynaptic potentials C. Synaptic vesicle release D. Myelin conduction

2. Alpha waves have a frequency range of:

A. 0.5-4 Hz B. 4-8 Hz C. 8-13 Hz D. 13-30 Hz

3. Alpha rhythm is best seen in which state?

A. Deep sleep B. Awake with eyes open C. Awake relaxed with eyes closed D. REM sleep

4. Standard EEG electrode placement system is called:

A. 20-40 system B. 10-20 system C. 5-10 system D. 25-50 system

5. Alpha block occurs when:

A. Eyes are closed B. Person becomes mentally inactive C. Eyes are opened D. During REM sleep

6. Beta waves are dominant during:

A. Deep sleep B. Light sleep C. Alert mental activity D. Meditation

7. Frequency of beta rhythm is:

A. 0.5-4 Hz B. 4-8 Hz C. 8-13 Hz D. 13-30 Hz

8. Presence of delta waves in an awake adult suggests:

A. Normal state B. Hyperventilation C. Organic brain disease D. Meditation

9. Theta waves are normally seen in:

A. Adults during wakefulness B. Infants and children C. REM sleep D. Epilepsy only

10.  A focal seizure typically shows:

A. 3 Hz generalized waves B. Localized spikes or sharp waves C. Beta wave excess D. Delta suppression

Appendix D

The delayed assessment questionnaire used during the crossover intervention is provided in Appendix D.

Crossover Intervention: Delayed Assessment (10 MCQ)

Q1. Presence of delta waves in an awake adult suggests:

A. Normal state B. Hyperventilation C. Organic brain disease D. Meditation

Q2. The hallmark EEG pattern of absence seizures is:

A. Polyspike bursts B. 3 Hz spike-and-wave C. Delta waves D. Beta bursts

Q3. Long-term meditation reduces:

A. Cortisol B. Melatonin C. Serotonin D. GABA

Q4. Meditation produces an increase in:

A. Alpha and beta activity B. Gamma activity C. Alpha and theta activity D. Delta activity

Q5. Odd-numbered electrodes denote:

A. Right side B. Midline C. Left side D. Occipital region

Q6. Electrode labeling "O1" indicates:

A. Central left B. Occipital left C. Occipital Right D. Frontal left

Q7. EEG signals require amplification because:

A. They are high voltage B. They are very low amplitude C. Brain tissue blocks signals D. Electrode gel interferes

Q8. Hyperventilation in children increases:

A. Beta waves B. Slow waves C. REM waves D. Gamma waves

Q9. EEG amplitude is typically:

A. 1-5 µV B. 5-10 µV C. 20-100 µV D. 200-500 µV

Q10. Presence of delta waves in an awake adult suggests:

A. Normal state B. Hyperventilation C. Organic brain disease D. Meditation

Appendix E

The structured feedback questionnaire used to evaluate students' perceptions regarding mind mapping as a self-directed learning tool is provided in Appendix E.

Feedback Questionnaire Based on a Five-Point Likert Scale

Name: Age: Gender:

1. Using mind maps improved my understanding of physiology concepts.

· Strongly disagree · Disagree · Neutral · Agree · Strongly agree

2. I felt more engaged in the lecture content when I have to make mind maps.

· Strongly disagree · Disagree · Neutral · Agree · Strongly agree

3. Mind maps helped me organize physiology concepts better.

· Strongly disagree · Disagree · Neutral · Agree · Strongly agree

4. Preparing mind maps encouraged active participation in active learning.

· Strongly disagree · Disagree · Neutral · Agree · Strongly agree

5. Using mind maps made me feel more confident about my knowledge of the lecture material.

· Strongly disagree · Disagree · Neutral · Agree · Strongly agree

6. Mind maps made the learning experience more enjoyable.

· Strongly disagree · Disagree · Neutral · Agree · Strongly agree

7. Mind maps encouraged me to engage in independent learning outside of lectures.

· Strongly disagree · Disagree · Neutral · Agree · Strongly agree

8. Using mind maps reduced the time I needed to study and understand physiology topics.

· Strongly disagree · Disagree · Neutral · Agree · Strongly agree

9. Mind maps were an effective tool for preparing for tests and exams.

· Strongly disagree · Disagree · Neutral · Agree · Strongly agree

10. I would be likely to use mind maps in subjects outside of physiology for similar benefits.

· Strongly disagree · Disagree · Neutral · Agree · Strongly agree
